# StemLoop-Finder: a Tool for the Detection of DNA Hairpins with Conserved Motifs

**DOI:** 10.1128/MRA.00424-21

**Published:** 2021-07-01

**Authors:** Alyssa A. Pratt, Ellis L. Torrance, George W. Kasun, Kenneth M. Stedman, Ignacio de la Higuera

**Affiliations:** a Department of Biology, Center for Life in Extreme Environments, Portland State University, Portland, Oregon, USA; b Department of Biochemistry and Biophysics, Oregon State University, Corvallis, Oregon, USA; c Department of Computer Science, Oregon State University, Corvallis, Oregon, USA; d Department of Biology, University of North Carolina Greensboro, Greensboro, North Carolina, USA; DOE Joint Genome Institute

## Abstract

Nucleic acid secondary structures play important roles in regulating biological processes. StemLoop-Finder is a computational tool to recognize and annotate conserved structural motifs in large data sets. The program is optimized for the detection of stem-loop structures that may serve as origins of replication in circular replication-associated protein (Rep)-encoding single-stranded (CRESS) DNA viruses.

## ANNOUNCEMENT

Circular replication-associated protein (Rep)-encoding single-stranded (CRESS) DNA viruses are a highly diverse group of viruses that includes several virus families, such as the *Circoviridae*, *Nanoviridae*, and *Geminiviridae* (1, 2). CRESS DNA viruses replicate through a rolling circle mechanism (3, 4). To initiate replication, the viral Rep nicks a conserved nonanucleotide sequence within a stem-loop DNA structure (5–9). Locating this feature is important for understanding the characteristics of a particular CRESS genome (10–13). Detection of potential stem-loop structures with nonanucleotide motifs was previously performed manually (10, 14). This process is time-consuming, especially for large metagenomic data sets. By automating identification of the nonanucleotide motifs and secondary structures, StemLoop-Finder increases efficiency and produces an annotated file with scored potential stem-loops for each viral genome analyzed. The biological significance of the predicted stem-loop structures should be assessed rationally or experimentally by the user.

**FIG 1:**
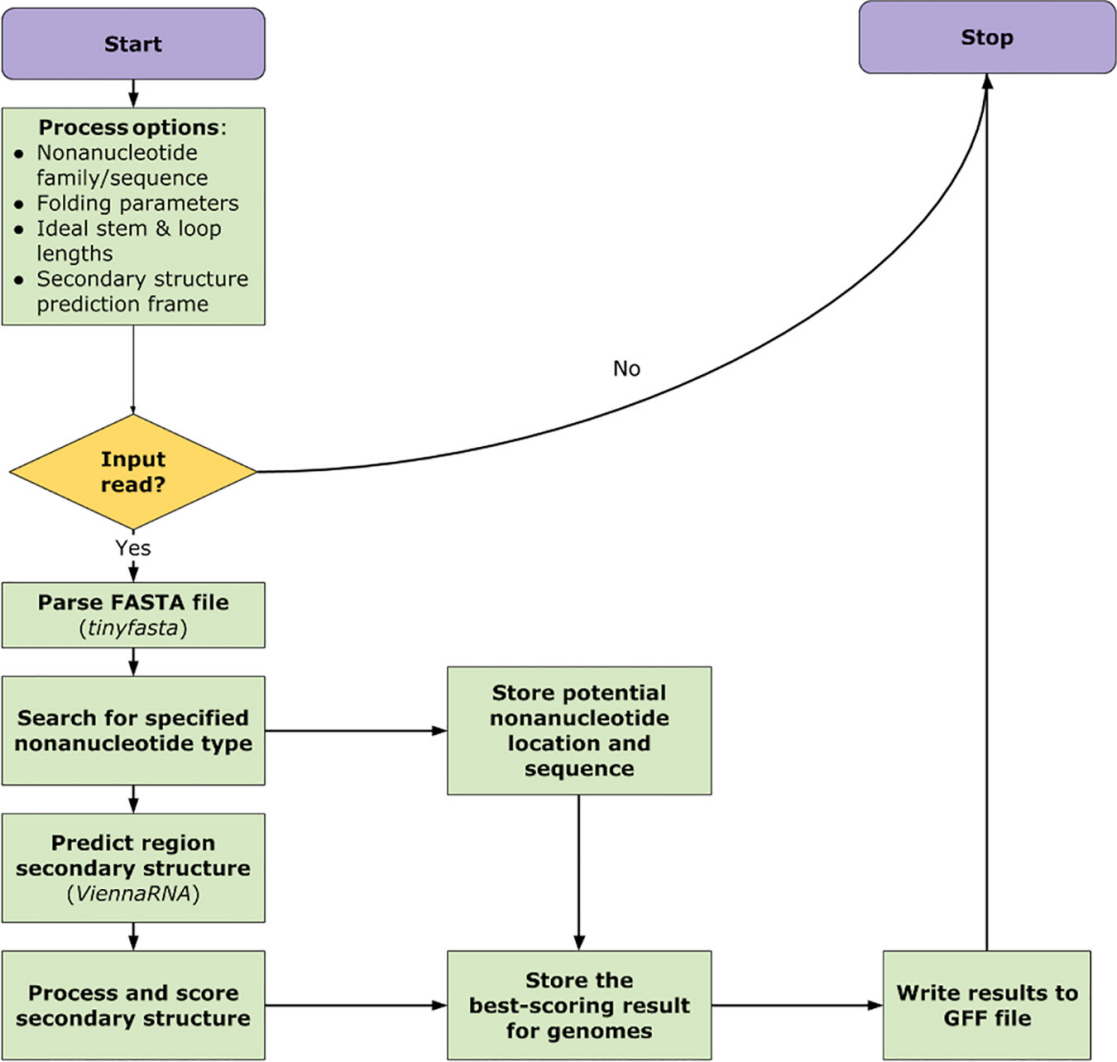
Flow chart depicting the StemLoop-Finder pipeline, with third-party tools indicated by italicized text.

StemLoop-Finder is written in Python within the PyCharm integrated development environment and can be run through the command-line interface on Mac OS, Windows (virtual machine), or Linux operating systems. It uses the ViennaRNA 2.0 library (15) to predict secondary structures in a DNA sequence using user-supplied prediction parameters and the library’s minimum free energy algorithms. It reads FASTA (with tinyfasta 0.1.0; https://pypi.org/project/tinyfasta/) and general feature format (GFF) sequence files and outputs stem-loop annotations as a GFF file and a more detailed comma-separated value (CSV) file (Fig. 1). Users input a desired CRESS DNA virus family or a 9-nucleotide sequence following the International Union of Pure and Applied Chemistry (IUPAC) degenerate base symbol standard (16). Another argument is used to determine the number of bases on either side of the nonanucleotide processed by the software for secondary structure prediction. These and other arguments are interpreted in Python with the argparse library.

ViennaRNA is used to predict the secondary structure of the defined region according to the parameters given, generating a dot-bracket model of the predicted structure (15). The user may use multiple parameter files and frame sizes to increase the number of stem-loop detections. In order to be scored, a stem-loop must have a stem length of at least 5 nucleotides and a loop length of at least 7 nucleotides. Each putative stem-loop is scored +1 point for each deviation of 1 nucleotide from the ideal stem or loop length and −5 points for high similarity to a specific nonanucleotide sequence, determined by the user as an argument or by the input viral family name. In order for a stem-loop to be annotated within the GFF file, it must have a score of less than 15 (or another user-defined value) and cannot have a nonanucleotide within 4 bases of the start or end of the potential stem-loop structure.

StemLoop-Finder was tested with a diverse set of publicly available CRESS DNA viral sequences from terrestrial arthropods for which stem-loops had been manually annotated (10). StepLoop-Finder detected stem-loops in 33 of the 44 sequences using the nonanucleotide motif NANTATTAC, which was used for the manual search (10). In six that were not detected, the nonanucleotide found manually did not fit NANTATTAC, and in the remaining five, the sequence surrounding the putative nonanucleotides was not predicted to form a stem-loop structure. Thus, StemLoop-Finder can be reliably used to automatically predict stem-loop structures in genomic and metagenomic data sets (12).

### Data availability.

The software source code is available on a public Bitbucket repository (https://bitbucket.org/crucicrew/sl-finder/src/master/) to be compiled from source or as a Docker container. It will remain freely available for the next 10 years alongside instructions for use and any applicable updates.
